# PALIPERIDONE PALMITATE 6-MONTH FORMULATION FOR THE TREATMENT OF SCHIZOPHRENIA: A 4-MONTH FOLLOW-UP STUDY

**DOI:** 10.1192/j.eurpsy.2023.2308

**Published:** 2023-07-19

**Authors:** S. L. Romero Guillena, G. Rodriguez Menendez, A. I. Florido Puerto, A. S. Fernández Flores

**Affiliations:** 1 UGC Salud Mental Virgen Macarena, Psychiatry, Seville, Spain

## Abstract

**Introduction:**

Relapse prevention is critical because psychopathology and functionality can worsen in patients with schizophrenia because the repeated episodes and we have strong evidence of antipsychotics efficacy for relapse prevention, but nonadherence rates in patients with schizophrenia are very high, even in comparison with other illness. The literature speaks of average rates of 42% in schizophrenia. For that, long-acting injectable antipsychotics (LAIs) are considered important treatment option, but they are underutilized (Taipale et al. Schizophrenia Bulletin 2017; 44, 1381–1387) (Garcia et al. J Clin Psychopharmacol 2016; 36(4)355-371).

There is extensive clinical trial evidence for the use of paliperidone palmitate 1-month (PP1M) and paliperidone palmitate 3-month (PP3M) formulations for maintaining treatment continuity and preventing relapses and risk of hospitalizations in patients with schizophrenia. (Najarian et al. Int J Neuropsychopharmacol 2022; 25(3) 238-251).

Paliperidone palmitate 6-month (PP6M) formulation is a presentation that provides a dosing interval of once every six months. It is the first and only antipsychotic to be administered twice a year.

**Objectives:**

The principal aim of this study was to evaluate the effectiveness, safety, and tolerability of the PP6M in people with non-acute schizophrenia in a naturalistic psychiatric outpatient setting

**Methods:**

Sample: 22 patients diagnosed with schizophrenia (DSM 5 criteria) that started treatment with PP6M after being stabilized with PP1M (N:10) or PP3M (N:12) (the treatment dose was not changed in the four months before study inclusion)

The mean dose of PP6M was 822.727 mg

Bimonthly, the following evaluations were performed during a follow-up period of 4 months:

The Clinical Global Impression-Schizophrenia scale (CGI-SCH)

Treatment adherence, concomitant medication, adverse events and the number of hospitalizations and emergency visits

Efficacy values: Percentage of patients who remained free of admissions at the end of 4months of follow-up.

Other evaluation criteria: Percentage of patients who never visited the emergency department at the end of 4 months of follow-up, average change from baseline visit to the final evaluation as assessed by score obtained on the following scale: GSI-SCH, treatment adherence rate and tolerability.

**Results:**

The percentage of patients who remained free of admissions at the end of the 4 months was 100% and the percentage of patients who never visited the emergency department at the end of 4 months was 100 %

Mean variations from baseline scores at 4 months were: (-0.21 ±0.31) on the GCI-SCH.

The rate of adherence to treatment with PP6M after 4 months was 100%.

Tolerability was good. None of the patients experienced an adverse event.

**Conclusions:**

In our study, we found that short-term treatment with paliperidone palmitate 6-month formulation is effective and well tolerated in clinical practice conditions

**Disclosure of Interest:**

None Declared

